# Tat-Endophilin A1 Fusion Protein Protects Neurons from Ischemic Damage in the Gerbil Hippocampus: A Possible Mechanism of Lipid Peroxidation and Neuroinflammation Mitigation as Well as Synaptic Plasticity

**DOI:** 10.3390/cells10020357

**Published:** 2021-02-09

**Authors:** Hyo Young Jung, Hyun Jung Kwon, Woosuk Kim, In Koo Hwang, Goang-Min Choi, In Bok Chang, Dae Won Kim, Seung Myung Moon

**Affiliations:** 1 Department of Anatomy and Cell Biology, Research Institute for Veterinary Science, College of Veterinary Medicine, Seoul National University, Seoul 08826, Korea; hyoyoung@snu.ac.kr (H.Y.J.); vetmed2@snu.ac.kr (I.K.H.); 2 Department of Biochemistry and Molecular Biology, Research Institute of Oral Sciences, College of Dentistry, Gangneung-Wonju National University, Gangneung 25457, Korea; donuts25@gwnu.ac.kr; 3 Department of Biomedical Sciences, Research Institute for Bioscience and Biotechnology, Hallym University, Chuncheon 24252, Korea; tank3430@hallym.ac.kr; 4 Department of Thoracic and Cardiovascular Surgery, Chuncheon Sacred Heart Hospital, College of Medicine, Hallym University, Chuncheon 24253, Korea; gmchoi@hallym.or.kr; 5 Department of Neurosurgery, Hallym University Sacred Heart Hospital, College of Medicine, Hallym University, Anyang 14068, Korea; nscib71@hallym.or.kr; 6 Department of Neurosurgery, Dongtan Sacred Heart Hospital, College of Medicine, Hallym University, Hwaseong 18450, Korea; 7 Research Institute for Complementary & Alternative Medicine, Hallym University, Chuncheon 24253, Korea

**Keywords:** endophilin A1, oxidative stress, ischemia, lipid peroxidation, synaptic plasticity

## Abstract

The present study explored the effects of endophilin A1 (SH3GL2) against oxidative damage brought about by H_2_O_2_ in HT22 cells and ischemic damage induced upon transient forebrain ischemia in gerbils. Tat-SH3GL2 and its control protein (Control-SH3GL2) were synthesized to deliver it to the cells by penetrating the cell membrane and blood–brain barrier. Tat-SH3GL2, but not Control-SH3GL2, could be delivered into HT22 cells in a concentration- and time-dependent manner and the hippocampus 8 h after treatment in gerbils. Tat-SH3GL2 was stably present in HT22 cells and degraded with time, by 36 h post treatment. Pre-incubation with Tat-SH3GL2, but not Control-SH3GL2, significantly ameliorated H_2_O_2_-induced cell death, DNA fragmentation, and reactive oxygen species formation. SH3GL2 immunoreactivity was decreased in the gerbil hippocampal CA1 region with time after ischemia, but it was maintained in the other regions after ischemia. Tat-SH3GL2 treatment in gerbils appreciably improved ischemia-induced hyperactivity 1 day after ischemia and the percentage of NeuN-immunoreactive surviving cells increased 4 days after ischemia. In addition, Tat-SH3GL2 treatment in gerbils alleviated the increase in lipid peroxidation as assessed by the levels of malondialdehyde and 8-iso-prostaglandin F2α and in pro-inflammatory cytokines such as tumor necrosis factor-α, interleukin-1β, and interleukin-6; while the reduction of protein levels in markers for synaptic plasticity, such as postsynaptic density 95, synaptophysin, and synaptosome associated protein 25 after transient forebrain ischemia was also observed. These results suggest that Tat-SH3GL2 protects neurons from oxidative and ischemic damage by reducing lipid peroxidation and inflammation and improving synaptic plasticity after ischemia.

## 1. Introduction

A plethora of animal models have been used for ischemic studies; however, Mongolian gerbils are generally preferred because of the absence of caudal communicating arteries between the vertebral and internal carotid arteries [1]. Simple clamping of common carotid arteries causes neuronal death in the hippocampus, thalamus, and neocortex within 2–4 days after ischemia [2,3]. This model shows high reproducibility, success, and survival rates [4]. Transient forebrain ischemia characterized by interruption of blood supply to the brain, is a leading cause of morbidity and mortality worldwide [5]. Interruption of blood supply rapidly depletes neuronal ATP, but subsequent reperfusion of blood flow markedly increases the formation of reactive oxygen species (ROS) and intracellular transport of Ca^2+^ [6,7]. In addition, transient forebrain ischemia increases pro-inflammatory cytokine release from astrocytes and microglia to enhance neuronal damage in the hippocampus [8,9]. Several mechanisms have been proposed for the execution of neuronal damage upon ischemic insult [10,11,12]; however, there is a dearth of therapeutic agents owing to their limited ability to cross the blood–brain barrier as well as the cell membrane. To overcome these difficulties, cell-penetrating peptides (CPPs) have been introduced [13], and one of these CPPs consists of the *trans*-acting activator of transcription (Tat), originating from the human immunodeficiency virus. This peptide has been depicted to translocate large proteins (up to 120 kDa) [14], DNA phages [15], and liposomes [16] across cell membranes. In previous studies, we and our colleagues demonstrated that Tat-cargo fusion protein could be delivered into the gerbil brain [17] and rabbit spinal cord [18].

Endophilin is a highly conserved protein that plays a role in the recycling of synaptic vesicles. It consists of two functional domains: N-terminal Bin–Amphiphysin–Rvs and C-terminal Src homology 3 (SH3). The C-terminal domain robustly binds to endocytic proteins such as dynamin, intersectin, and synaptojanin [19,20,21,22,23]. Endophilins are encoded by three genes in vertebrates of which endophilin A1 is exclusively expressed in the brain [24,25,26], while endophilin A2 is ubiquitously expressed and endophilin A3 is observed in the brain and testis [27]. In the hippocampus, endophilin A family proteins are expressed in the pre- and post-synaptic terminals [28,29], and knockout of the individual endophilin A family does not cause any problems in life span. However, deletion of endophilin A1 and A2 or all endophilin A families results in neurodegeneration and perinatal lethality, respectively [22,30]. Endophilin A1 is highly expressed in the hippocampal CA1 and CA3 regions, and deletion of endophilin A1 and A2 shows significant decrease in spine size in these hippocampal regions [31].

Several studies have demonstrated conflicting evidence on the effects of endophilin A1 during discrete diseases. Endophilin A1 levels are significantly increased in the brain of individuals with Alzheimer’s disease [32] and temporal lobe epilepsy [33]. In addition, the susceptibility and severity of epilepsy are mitigated by the knockdown of endophilin A1 in mice [33]. Further, endophilin A1 could regulate brain-derived neurotrophic factor and the tropomyosin receptor kinase B signaling pathway to mediate hippocampal plasticity [29,34,35]. In addition, miR-330 inhibited endophilin A1 expression by directly binding to the 3′-UTR of *SH3GL2* [36,37], and miR-330 antagomir treatment decreased neuronal damage 6 h after middle cerebral artery occlusion [38]. However, there are few studies that detail the changes of SH3GL2 expression after transient forebrain ischemia in the gerbil hippocampus and the effects of endophilin A1 against oxidative damage in HT22 cells and against ischemic damage in the gerbil hippocampus.

In the present study, we examined the chronological changes of SH3GL2 immunoreactivity in the gerbil hippocampus after transient forebrain ischemia and synthesized the Tat-endophilin A1 fusion protein (Tat-SH3GL2) to elucidate its effects and role against oxidative and ischemic damage in HT22 cells and gerbil hippocampus, respectively.

## 2. Materials and Methods

### 2.1. Synthesis of Tat-SH3GL2 and Its Efficient Delivery into HT22 Cells

Tat-endophilin A1 was synthesized by cloning human endophilin A1 cDNA in a TA vector and Tat-1 expression vector. To visualize and compare the effects of endophilin A1 with and without the Tat-1 expression vector, the expression vector of endophilin A1 was constructed with a polyhistidine tag. Tat-SH3GL2 and Control-SH3GL2 plasmids were amplified and purified proteins were obtained as described previously [17,18]. Purified proteins were confirmed by Western blot analysis using polyhistidine antibody (1:3000, Sigma, St. Louis, MO, USA) wherein the tagged protein was detected with chemiluminescent reagent as per the manufacturer’s instructions (Amersham, Franklin Lakes, NJ, USA).

Different concentrations of Tat-SH3GL2 and Control-SH3GL2 (0.5 to 5.0 μM) were incubated over a period of time (15 to 60 min) with 3 μM protein to observe the time- and concentration-dependent delivery of protein into HT22 cells. In addition, Tat-SH3GL2 was incubated for 60 h to elucidate the intracellular stability and degradation of Tat-SH3GL2 in HT22 cells. Intracellular delivery was confirmed by Western blot analysis using the specific antibody against the target protein as described previously [17,18].

### 2.2. Confirmation of Intracellular Delivery of Tat-SH3GL2 into HT22 Cells and Gerbil Hippocampus

Intracellular delivery was visualized by immunocytochemistry using a polyhistidine antibody. Briefly, HT22 cells were incubated with 3 μM Tat-SH3GL2 and Control-SH3GL2 proteins for 60 min and subsequently fixed with 4% paraformaldehyde for 5 min at 25 °C. Cells were sequentially incubated with mouse anti-polyhistidine primary antibody (1:2000, Sigma) and Alexa Fluor^®^ 488-conjugated anti-mouse IgG secondary antibody (1:1000; Jackson ImmunoResearch, West Grove, PA, USA). The nuclei were stained with 1 μg/mL 4,6-diamidino-2-phenylindole (DAPI, Thermo Fisher Scientific, Waltham, MA, USA). Immunofluorescence images were obtained with a confocal fluorescence microscope (LSM 510 META NLO; Zeiss GmbH, Jena, Germany). To ensure the internalization of Tat-SH3GL2 and not just localization on the outer surface of the cells, Western blot analysis for polyhistidine was performed in cell lysates and aspirated media in Tat-SH3GL2 treated HT22 cells.

Delivery of Tat-SH3GL2 and Control-SH3GL2 was assessed by immunohistochemical staining for polyhistidine. Briefly, gerbils (*n* = 5 in each group) received intraperitoneal injection of vehicle, Control-SH3GL2 (4 mg/kg), or Tat-SH3GL2 (4 mg/kg) and animals were anesthetized with an intraperitoneal injection of 75 mg/kg alfaxalone (Careside, Seongnam, South Korea) and 10 mg/kg xylazine (Bayer Korea, Seoul, South Korea) 8 h after Control-SH3GL2 or Tat-SH3GL2 treatment. Animals were perfused transcardially with physiological saline and 4% paraformaldehyde. Coronal sections (30-μm thickness) were made between 2.0 and 2.7 mm caudal to the bregma regions based on a gerbil brain atlas [39]. The sections were incubated with mouse anti-polyhistidine primary antibody (1:2000, Sigma) and Cy3-conjugated anti-mouse IgG secondary antibody (1:600; Jackson ImmunoResearch).

### 2.3. Effect of Tat-SH3GL2 on H_2_O_2_-Induced Oxidative Stress in HT22 Cells

HT22 cells were incubated with different concentrations (0.5 to 5.0 µM) of Control-SH3GL2 and Tat-SH3GL2 for 60 min, and subsequently the cells were exposed to 1 mM H_2_O_2_ treatment for 5 h to ascertain the optimal concentration of proteins for neuroprotection against oxidative stress. Thereafter, the cells were harvested, and the cellular damage was assessed by the water-soluble tetrazolium salt-1 (WST-1) assay kit (Abcam, Cambridge, UK) as per the manufacturer’s protocol.

DNA fragmentation was confirmed by terminal deoxynucleotidyl transferase-mediated biotinylated dUTP nick end labeling (TUNEL) assay as described in previous studies [17,18]. Briefly, HT22 cells were incubated with 5.0 µM Control-SH3GL2 and Tat-SH3GL2 for 60 min and thereafter the cells were exposed to 1 mM H_2_O_2_ for 3 h. The cells were then fixed with 4% paraformaldehyde and TUNEL staining was conducted according to the manufacturer’s protocol (Sigma-Aldrich, St. Louis, MO, USA).

ROS formation was visualized by 2,7-dichlorofluorescein (DCF) fluorescence as described in previous studies [17,18]. Briefly, cells were treated with 5.0 µM Control-SH3GL2 and Tat-SH3GL2 for 60 min, followed by 1 mM H_2_O_2_ for 10 min, and 20 μM DCF diacetate (DCF-DA, Abcam) for 30 min to convert DCF-DA to DCF by ROS. Thereafter, the cells were fixed with 4% paraformaldehyde and DCF fluorescence was visualized using a fluorescence microscope (Nikon Eclipse 80i, Tokyo, Japan). The fluorescence intensities were quantified using a Fluoroskan enzyme-linked immunosorbent assay (ELISA) plate reader (Labsystems Oy, Helsinki, Finland).

### 2.4. Changes of SH3GL2 Immunoreactivity in the Gerbil Hippocampus after Ischemia

Mongolian gerbils (male, 3-month old) were purchased from Japan SLC Inc. (Shizuoka, Japan) and the experimental protocols were approved by the Institutional Animal Care and Use Committee (IACUC) of the Seoul National University, Seoul, Korea (SNU-190516-7). Transient forebrain ischemia in gerbils was induced by occlusion of both the common carotid arteries for 5 min under anesthesia with 2.5% isoflurane (Hana Pharm Co., Ltd., Hwaseong, South Korea), as described in previous studies [40,41]. Complete occlusion of both the arteries was confirmed by observing the central artery of the retina using an ophthalmoscope (HEINE K180^®^, Heine Optotechnik, Herrsching, Germany). Animals were re-anesthetized with intraperitoneal injection of 75 mg/kg alfaxalone and 10 mg/kg xylazine and transcardiac perfusion was performed as described above. Coronal sections (30-μm thickness) were made between 2.0 and 2.7 mm caudal to the bregma regions based on a gerbil brain atlas [39]. Seven sections per animal that were obtained from regions separated from each other by at least 90 μm, were selected and immunohistochemical staining for SH3GL2 was performed using rabbit anti-SH3GL2 (1:200; Synaptic Systems, Göttingen, Germany), biotinylated goat anti-rabbit IgG (1:200; Vector, Burlingame, CA, USA), and peroxidase-conjugated streptavidin (Vector). Sections were visualized upon reaction with 3,3′-diaminobenzidine tetrachloride (Sigma).

### 2.5. Effects of Tat-SH3GL2 against Brain Ischemic Damage in Gerbils

Transient forebrain ischemia was induced as described above and animals received intraperitoneal injection of Tat peptide (4 mg/kg), Control-SH3GL2 (4 mg/kg), or Tat-SH3GL2 (4 mg/kg) 30 min after reperfusion. A day after ischemia/reperfusion, spontaneous motor activity was recorded with a digital camera system (Basler106200, Ahrensburg, Germany) for 60 min to assess the amelioration of motor activity induced by hippocampal damage after ischemia [42]. The recorded observations were analyzed by Ethovision XT14 (Noldus, Wageningen, The Netherlands), as described in previous studies [17,43].

Four days after ischemia, animals were anesthetized with intraperitoneal injection of 75 mg/kg alfaxalone and 10 mg/kg xylazine and transcardiac perfusion was conducted as described above. Coronal sections (30-μm thickness) were made between 2.0 and 2.7 mm caudal to the bregma regions based on a gerbil brain atlas [39]. Five sections per animal that were obtained from regions separated from each other by at least 120 μm, were selected. Immunohistochemical staining for NeuN to detect mature neurons in the hippocampus was performed as described in previous studies [17,40,41,43]. Briefly, antibodies used in the present study were mouse anti-NeuN antibody (1:1000; EMD Millipore, Temecula, CA, USA) and biotinylated goat anti-mouse IgG (1:200; Vector,). Sections were visualized upon reaction with 3,3′-diaminobenzidine tetrachloride (Sigma).

### 2.6. Mechanisms of Tat-SH3GL2 against Brain Ischemic Damage in Gerbils

Tat peptide (4 mg/kg), Control-SH3GL2 (4 mg/kg), and Tat-SH3GL2 (4 mg/kg) were administered in gerbils 30 min after ischemic surgery. Subsequently, the animals were sacrificed 3 h and 12 h after ischemia/reperfusion to assess malondialdehyde (MDA) and 8-iso-prostaglandin F2α (8-iso-PGF2α) levels, which constitute the oxidative products from polyunsaturated fatty acid and arachidonic acid, respectively [44,45]. Animals were sacrificed 6 h after ischemia/reperfusion to measure the pro-inflammatory cytokines such as tumor necrosis factor-α (TNF-α), interleukin (IL)-1β, and IL-6 in the hippocampus as described in a previous study [46]. In addition, animals were sacrificed 1 day and 4 days after ischemia/reperfusion to observe changes in the postsynaptic density 95 (PSD95), synaptophysin, and synaptosome associated protein 25 (SNAP-25), which have been implicated in synaptic transmission. Briefly, animals were sacrificed with 75 mg/kg alfaxalone, 10 mg/kg xylazine, and isoflurane, and the brain was removed from the skull. The hippocampal CA1 slice (500-μm thickness) was obtained using a vibratome (Leica Microsystems, Wetzlar, Germany) between 2.0 and 2.7 mm caudal to the bregma regions based on a gerbil brain atlas [39]. MDA (Cayman Chemical Company, Ann Arbor, MI, USA), 8-iso-PGF2α (Cayman Chemical Company), TNF-α (EMD Millipore, Billerica, MA, USA), IL-1β (EMD Millipore), and IL-6 (EMD Millipore) were measured using ELISA kits following the manufacturer’s instructions. PSD95, synaptophysin, and SNAP-25 levels were evaluated by Western blot analysis and the hippocampus sections were homogenized in Syn-PER Reagent (Thermo Fisher Scientific) using a tissue grinder. The homogenate was sequentially centrifuged at 1200× *g* for 10 min and 15,000× *g* for 20 min to separate the synaptosome fraction. Antibodies used in the present study were mouse anti-PSD95 antibody (1:500; Abcam), mouse anti-synaptophysin (1:500; Abcam), rabbit anti-SNAP25 (1:1000; Cell Signaling Technology, Danvers, MA, USA), peroxidase-conjugated anti-mouse IgG (Vector; 1:500), and peroxidase-conjugated anti-rabbit IgG (Vector; 1:500). The protein bands obtained through Western blotting were visualized with chemiluminescent reagents according to the manufacturer’s instructions (Amersham).

### 2.7. Data Quantification and Statistical Analysis

NeuN-immunoreactive structures in five sections at 120-µm intervals per animal were taken under 100× magnification in the mid-point of the hippocampal CA1 region. The number of NeuN-immunoreactive neurons was counted using OPTIMAS 6.5 software (CyberMetrics Corporation, Phoenix, AZ, USA) as described previously [41,43]. In addition, polyhistidine and endophilin A1 immunoreactivity was assessed based on its optical density and pixel numbers using ImageJ v. 1.80 software (National Institutes of Health, Bethesda, MD, USA), as described previously [40]. Data are presented as percent averages with standard deviation compared to the control group (which was set as 100%) and differences in averages were statistically analyzed by a one-way or two-way analysis of variance (ANOVA) followed by Bonferroni’s post-hoc test using GraphPad Prism 5.01 software (GraphPad Software, Inc., La Jolla, CA, USA).

## 3. Results

### 3.1. Construction of Tat-SH3GL2 and Control-SH3GL2 and Their Efficient Delivery to HT22 Cells

**Figure 1:**
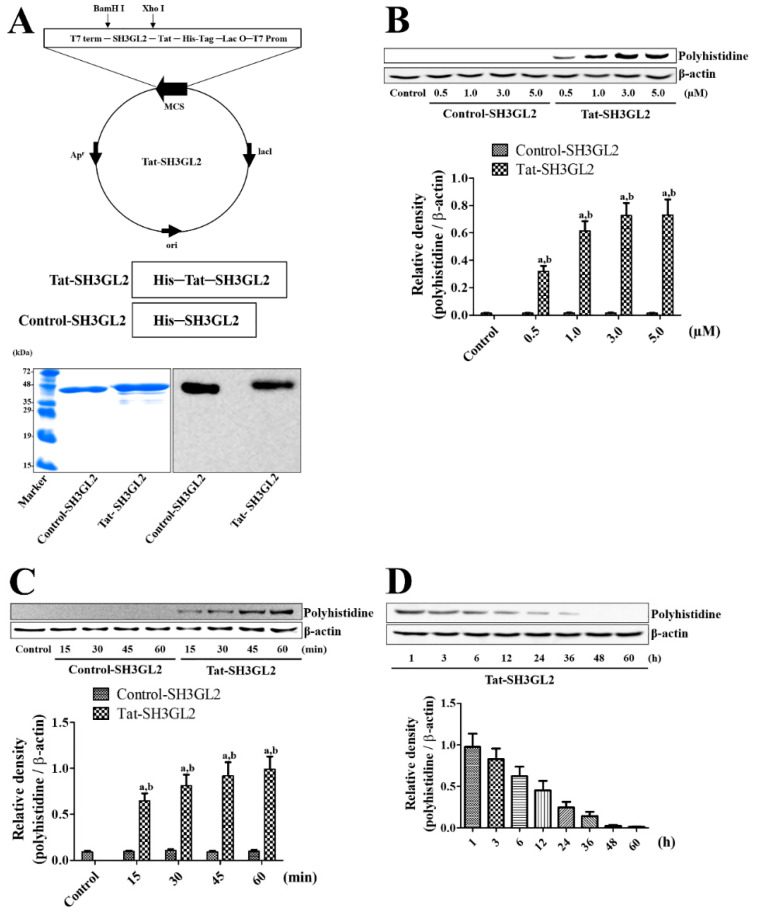
Construction and intracellular delivery of Tat-SH3GL2 and Control-SH3GL2. (**A**) Control-SH3GL2 and Tat-SH3GL2 vectors were constructed and expression was confirmed using Coomassie brilliant blue staining and Western blot analysis. (**B**) Concentration (0.5–5 μM) and (**C**) time-dependent (0–60 min) intracellular delivery of Tat-SH3GL2 and Control-SH3GL2 treatment in HT22 cells was assessed by Western blot analysis (**D**) Time-dependent degradation of the delivered proteins was evaluated using Western blot analysis (**B**,**C**) Significance in the intensity of polyhistidine bands was analyzed by two-way ANOVA followed by a Bonferroni’s post-hoc test (a, *p* < 0.05, significantly different from the control group; b, *p* < 0.05, significantly different from the Control-SH3GL2-treated group).

Protein synthesis of Tat-SH3GL2 and Control-SH3GL2 was assessed by Coomassie brilliant blue staining and Western blot analysis for polyhistidine to detect the His-Tag inserted in the vector. Clear bands of successfully synthesized Tat-SH3GL2 and Control-SH3GL2 were observed at 42.3 kDa and 40.7 kDa, respectively (Figure 1A). A concentration-dependent intracellular delivery of Tat-SH3GL2 was observed in HT22 cells after 60 min of protein incubation as polyhistidine bands were observed when 3.0 μM and 5.0 μM of Tat-SH3GL2 were utilized (Figure 1B). In addition, Tat-SH3GL2 incubation showed a time-dependent intracellular delivery, while Control-SH3GL2 incubation did not show any protein bands of polyhistidine at any concentration or time of protein incubation (Figure 1C). Intracellular stability and degradation of Tat-SH3GL2 was confirmed by polyhistidine bands, and intracellularly delivered Tat-SH3GL2 decreased in a time-dependent manner from 1 h to 36 h after incubation and was nearly undetectable thereafter (Figure 1D).

### 3.2. Intracellular Delivery of Tat-SH3GL2 into HT22 Cells and Gerbil Hippocampus

**Figure 2:**
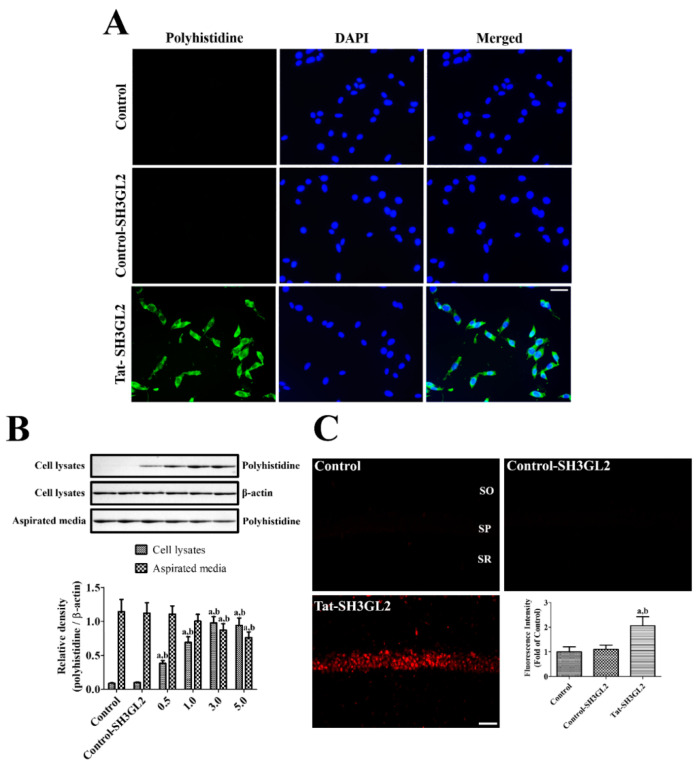
Confirmation of intracellular delivery of Tat-SH3GL2 and Control-SH3GL2. (**A**) Intracellularly delivered Tat-SH3GL2 and Control-SH3GL2 proteins were visualized in HT22 cells by immunocytochemical staining for polyhistidine. Scale bar = 20 μm. (**B**) Confirmation of concentration (0.5–5 μM)-dependent intracellular delivery of Tat-SH3GL2 and Control-SH3GL2 treatment in cell lysates and aspirated media of HT22 cells was assessed by Western blot analysis. (**C**) Hippocampal delivery was visualized in gerbils by immunohistochemical staining for polyhistidine 8 h after Tat-SH3GL2 and Control-SH3GL2 treatment. Scale bar = 50 μm. (**B**,**C**) Bar graph represents the mean ± standard deviation. Significance in the intensity of the polyhistidine bands and polyhistidine immunofluorescence was analyzed by one-way ANOVA followed by a Bonferroni’s post-hoc test (a, *p* < 0.05, significantly different from the control group; b, *p* < 0.05, significantly different from the Control-SH3GL2-treated group).

In HT22 cells, the intracellular delivery of Tat-SH3GL2 was visualized by immunocytochemical staining for polyhistidine. In the control experiment, incubation of HT22 cells with 3.0 μM Control-SH3GL2 for 60 min did not display any polyhistidine immunoreactive cells, whereas incubation with 3.0 μM Tat-SH3GL2 for 60 min showed strong polyhistidine immunoreactive cells in HT22 cells (Figure 2A).

In Tat-SH3GL2-treated HT22 cells, internalization of Tat-SH3GL2 was confirmed by Western blot analysis for polyhistidine in cell lysates and aspirated media. Incubation with Tat-SH3GL2 for 60 min showed concentration-dependent increases of polyhistidine bands in cell lysates, and the polyhistidine levels were significantly higher in the Tat-SH3GL2-treated group compared to that in the control or Control-SH3GL2-treated group. In contrast, polyhistidine bands were decreased in a concentration-dependent manner and showed significantly lower levels in the range of 3.0 to 5.0 μM in the Tat-SH3GL2-treated group compared to that in the control or Control-SH3GL2-treated group (Figure 2B).

Delivery of Tat-SH3GL2 into the gerbil hippocampal CA1 region was assessed by immunohistochemical staining for polyhistidine. In the control group, polyhistidine immunoreactive structures were not detectable in the hippocampal CA1 region. In the Control-SH3GL2-treated group, polyhistidine immunoreactivity was very faintly detected in the hippocampal CA1 region and there were no significant differences in polyhistidine immunoreactivity between the control and Control-SH3GL2-treated groups. In the Tat-SH3GL2-treated group, polyhistidine immunoreactive structures were mainly detected in the stratum pyramidale of the CA1 region and polyhistidine immunoreactivity was significantly increased to 2.06-fold that of the control group (Figure 2C).

### 3.3. Effect of Tat-SH3GL2 and Control-SH3GL2 against H_2_O_2_-Induced Oxidative Damage in HT22 Cells

**Figure 3:**
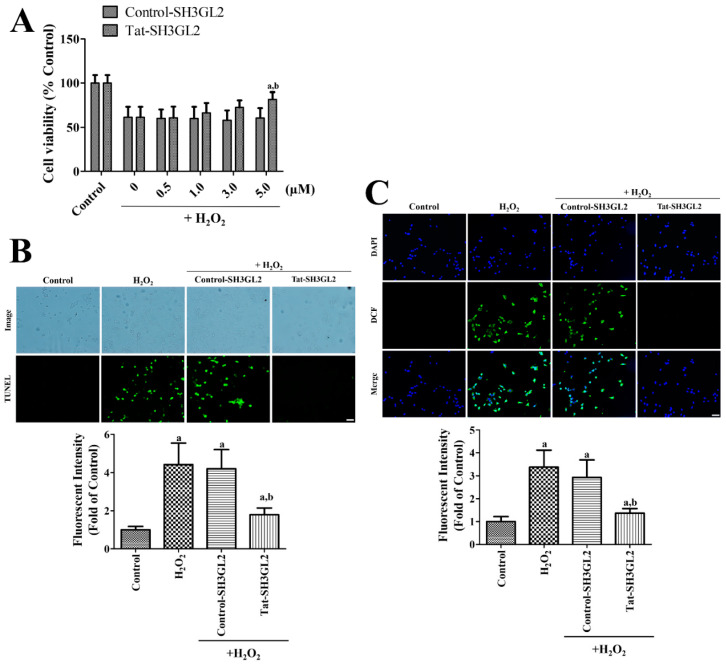
Neuroprotective effects of Tat-SH3GL2 and Control-SH3GL2 against oxidative stress induced by H_2_O_2_ in HT22 cells. (**A**) Optimal concentration of Tat-SH3GL2 and Control-SH3 to protect neurons from cell death induced by 1 mM H_2_O_2_ was determined by WST-1 assay. (**B**) DNA fragmentation in the cells was detected by TUNEL assay when the cells were treated with 5.0 μM Tat-SH3GL2 and Control-SH3GL2 and 1 mM H_2_O_2_ for 3 h. (**C**) Reactive oxygen species (ROS) formation in the cells was measured by DCF fluorescence upon pre-incubation with 5.0 μM Tat-SH3GL2 and Control-SH3GL2, 1 mM H_2_O_2_ for 10 min, and 20 μM DCF-DA for 30 min. (**B**,**C**) Scale bar = 50 μm. Intensities of TUNEL positive cells and DCF fluorescence were also measured. (**A**–**C**) Significances in the changes in cell viability and fluorescence intensities were analyzed by two-way or one-way ANOVA followed by a Bonferroni’s post-hoc test (a, *p* < 0.05, significantly different from the control group; b, *p* < 0.05, significantly different from the H_2_O_2_ alone group). Bar graph represents the mean ± standard deviation.

Cells were incubated with Tat-SH3GL2 or Control-SH3GL2 for 1 h and subsequently oxidative stress was induced by 1 mM H_2_O_2_ treatment for 5 h to assess the protective effects and optimal concentration of Tat-SH3GL2 against oxidative damage. In the 1 mM H_2_O_2_ only group, cell viability significantly decreased by 0.62-fold that of the control group. However, pre-incubation with Tat-SH3GL2 showed a concentration-dependent increase in cell viability and a significant increase (0.82-fold that of control group) in cell viability was only found in the 5 μM Tat-SH3GL2-treated group compared to that in the H_2_O_2_ only group and control-SH3GL2-treated group. In contrast, treatment with control-SH3GL2 did not show any significant effect on cell viability compared with the 1 mM H_2_O_2_ only group (Figure 3A).

Cell damage based on DNA fragmentation was assessed by TUNEL staining when HT22 cells were incubated with 5 μM Tat-SH3GL2 or Control-SH3GL2 for 1 h and then subjected to 1 mM H_2_O_2_ for 3 h. In the control group, very few TUNEL-positive cells were detected in the HT22 cells; while in the H_2_O_2_ only group and pre-incubated group with Control-SH3GL2, abundant TUNEL-positive cells were observed. In these groups, the fluorescence intensity significantly increased to 4.44- and 4.21-fold, respectively, when compared with that of the control group. In the cells that were pre-incubated with Tat-SH3GL2, a few TUNEL-positive cells were found and the fluorescence intensity was significantly decreased to 1.79-fold that of the control group when compared with that in the H_2_O_2_ only group and Control-SH3GL2-treated group (Figure 3B).

The generation of ROS at early time points upon H_2_O_2_ treatment was assessed by DCF fluorescence, which involved the conversion of DCF-DA when the HT22 cells were incubated with 5 μM Tat-SH3GL2 or Control-SH3GL2 for 1 h, followed by 1 mM H_2_O_2_ for 10 min, and then 20 μM DCF-DA for 30 min. In the control group, DCF fluorescence was not detectable in the HT22 cells, whereas in the H_2_O_2_ only group and pre-incubated group with Control-SH3GL2, strong DCF fluorescence was observed in the cytoplasm of HT22 cells. In these groups, the fluorescence intensity of DCF was significantly increased to 3.38- and 2.93-fold that of the control group, respectively. Pre-incubation with Tat-SH3GL2 revealed compromised DCF fluorescence in the HT22 cells, and the DCF fluorescence intensity significantly decreased by 1.37-fold that of the control group (Figure 3C).

### 3.4. Time-Dependent Changes of Endophilin A1 Immunoreactivity in the Hippocampus after Ischemia

**Figure 4:**
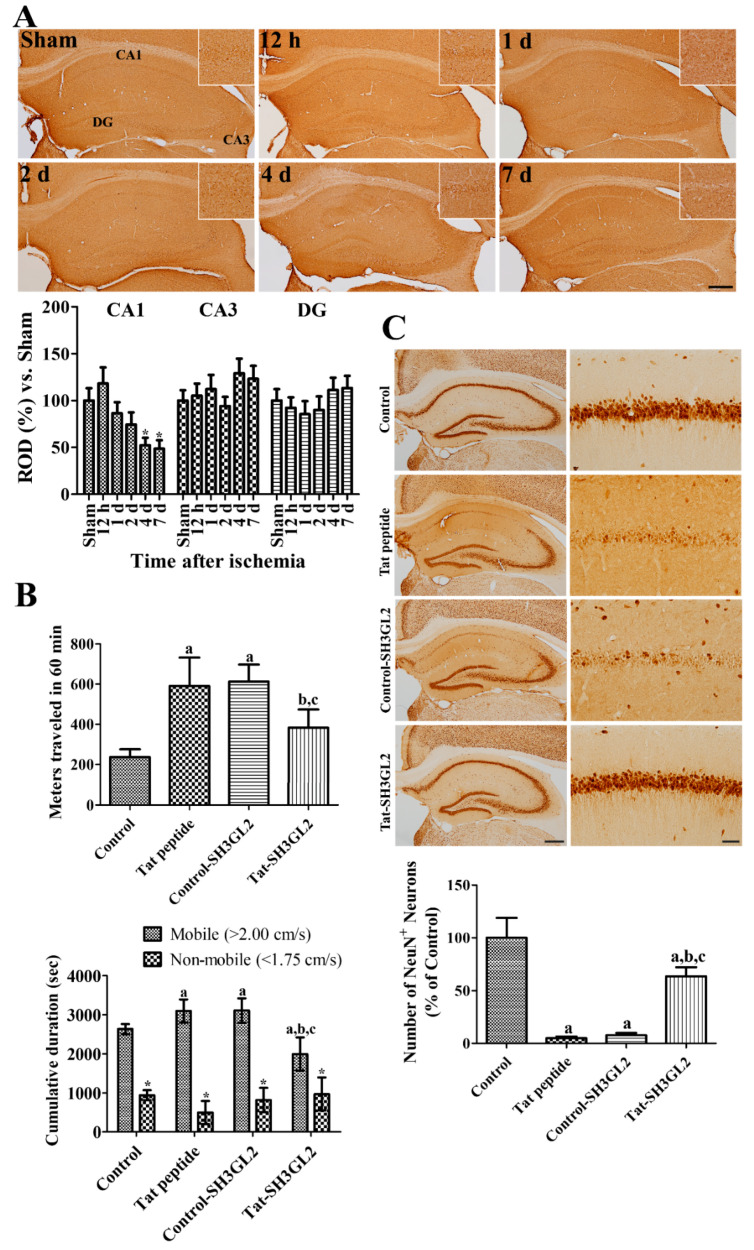
Changes of endophilin A1 immunoreactivity in the hippocampus and effect of Tat-SH3GL2 and Control-SH3GL2 on spontaneous motor activity and neuronal death in gerbils after ischemia. (**A**) Immunohistochemical staining for endophilin A1 was performed in the hippocampus including CA1, CA3, and the dentate gyrus (DG) at various time points after ischemia. Inset image shows highly magnified CA1. Scale bar = 400 μm. (**B**) Spontaneous motor activity was tracked, and the traveled distance and cumulative duration in mobile and non-mobile phases were calculated one day after ischemia in the sham-operated (control) group, Tat peptide (vehicle)-, Control-SH3GL2-, and Tat-SH3GL2-treated groups. (**C**) Immunohistochemical staining for NeuN was conducted in the hippocampus, and magnification was completed for the CA1 region in the control, Tat peptide-, Control-SH3GL2-, and Tat-SH3GL2-treated groups at 4 days after ischemia. Scale bar = 400 μm (hippocampus) and 50 μm (CA1 region). Significances in the difference of the endophilin A1 immunoreactivity, distance travelled, and the number of NeuN-immunoreactive neurons were analyzed by one-way ANOVA followed by a Bonferroni’s post-hoc test (*n* = 5 per group; *, *p* < 0.05, significantly different from the sham-operated group; a, *p* < 0.05, significantly different from the control group; b, *p* < 0.05, significantly different from the vehicle-treated group; c, *p* < 0.05, significantly different from the Control-SH3GL2-treated group). Bar graph represents the mean ± standard deviation.

In the sham-operated group, weak endophilin A1 immunoreactivity was found in the stratum pyramidale of the hippocampal CA1 and CA3 regions and in the granule cell layer and polymorphic layer of the dentate gyrus. Endophilin A1 immunoreactivity was slightly higher in the hippocampal CA1 region compared to that in the sham-operated group, while in the other regions endophilin A1 immunoreactivity was similarly observed. Thereafter, endophilin A1 immunoreactivity was decreased with time after ischemia in the hippocampal CA1 region by 7 days after ischemia, but it was maintained in the hippocampal CA3 and dentate gyrus. Endophilin A1 immunoreactivity showed significantly lower levels 4 and 7 days after ischemia compared to that in the sham-operated group (Figure 4A).

### 3.5. Effect of Tat-SH3GL2 and Control-SH3GL2 against Ischemic Injury in Gerbils

The neuroprotective effect of Control-SH3GL2 and Tat-SH3GL2 against ischemic damage was evaluated by analyzing the spontaneous motor activity of the animals one day after ischemia as hyperactivity was known to be induced by ischemic damage in the hippocampus. In the Tat peptide or Control-SH3GL2-treated group, traveled distance was significantly increased to 2.49- and 2.59-fold that of the control group, respectively. In addition, the cumulative duration in the mobile phase, but not the non-mobile phase, was significantly longer in the Tat peptide or Control-SH3GL2-treated group. In the Tat-SH3GL2-treated group, spontaneous motor activity was significantly decreased to 1.62-fold that of the control group and the cumulative duration in mobile phase was shortened significantly compared to those in control, Tat peptide, or Control-SH3GL2-treated groups, thereby displaying reduced motor activity when compared with the Tat peptide or Control-SH3GL2-treated groups (Figure 4B).

Neuronal death was confirmed by immunohistochemical staining for neuronal nuclei (NeuN), which is a marker for mature neurons. In the control group, NeuN-immunoreactive neurons were found in all hippocampal regions, including the CA1 region. In the Tat peptide or Control-SH3GL2-treated group, only a few NeuN-immunoreactive neurons were detected in the hippocampal CA1 region, and NeuN-immunoreactive neurons were abundantly observed in other hippocampal regions. In these groups, the number of NeuN-immunoreactive neurons was 0.05- and 0.08-fold that of the control group, respectively. In the Tat-SH3GL2-treated group, numerous NeuN-immunoreactive neurons were observed in the hippocampal CA1 region, and the number was significantly increased to 0.64-fold that in the control group compared to the Tat peptide or Control-SH3GL2-treated groups (Figure 4C).

### 3.6. Mechanisms of Tat-SH3GL2 and Control-SH3GL2 against Ischemic Injury in Gerbils

**Figure 5:**
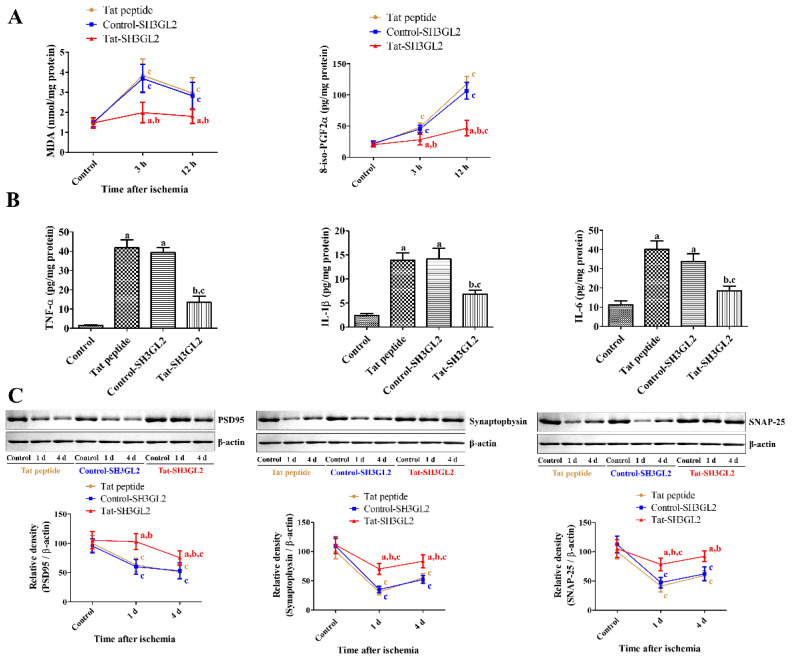
Effects of Tat-SH3GL2 and Control-SH3GL2 on lipid peroxidation, pro-inflammatory cytokine releases, and synaptic plasticity in the hippocampal CA1 region of gerbils. (**A**) Malondialdehyde (MDA) and 8-iso-PGF2α levels were measured using ELISA assay kits 3 h and 12 h after ischemia in Tat peptide-, Control-SH3GL2-, and Tat-SH3GL2-treated groups. (**B**) TNF-α, IL-1β, and IL-6 levels were determined using ELISA assay kits 6 h after ischemia. (**C**) PSD95, synaptophysin, and SNAP-25 protein levels were measured using Western blot 1 and 4 days after ischemia. (A and B) Data were analyzed by two-way ANOVA followed by a Bonferroni’s post-hoc test (*n* = 5 per group; a, *p* < 0.05, significantly different from the Tat peptide-treated group; b, *p* < 0.05, significantly different from the Control-SH3GL2-treated group; c, *p* < 0.05, significantly different from the control group). Bar graph represents the mean ± standard deviation.

Neuroprotective mechanisms of Tat-SH3GL2 and Control-SH3GL2 were evaluated by ELISA for MDA and 8-iso-PGF2α in the hippocampus at early time points after ischemia induction. In the Tat peptide- and Control-SH3GL2-treated groups, MDA levels were significantly elevated 3 h after ischemia and decreased 12 h post ischemia, although MDA levels were significantly higher than that of their respective controls. Concurrently, the levels of 8-iso-PGF2α within the hippocampus showed a steady and significant increase by 12 h post ischemia compared to that of their controls. In the Tat-SH3GL2-treated group, MDA and 8-iso-PGF2α levels showed minimal changes after ischemia induction and showed significantly lower levels at 3 h and 12 h after ischemia compared to those in the Tat peptide or Control-SH3GL2-treated group, respectively (Figure 5A).

TNF-α, IL-1β, and IL-6 levels were determined in the hippocampus using ELISA kit. In the Tat peptide- and Control-SH3GL2-treated groups, TNF-α, IL-1β, and IL-6 levels were significantly increased in the hippocampus 6 h after ischemia compared to those in the control group. In the Tat-SH3GL2-treated group, TNF-α, IL-1β, and IL-6 levels significantly decreased compared to those in the Tat peptide or Control-SH3GL2-treated groups (Figure 5B).

PSD95, synaptophysin, and SNAP-25 levels were assessed in the hippocampus by Western blot analysis because ischemia and Tat-SH3GL2 have been shown to affect synaptic plasticity in the hippocampus. In the Tat peptide- and Control-SH3GL2-treated groups, PSD95 protein levels gradually decreased 1 day and 4 days after ischemia, while synaptophysin and SNAP-25 levels increased 4 days after ischemia. In all the cases, the protein levels showed significantly lower levels compared to that of their controls. In the Tat-SH3GL2-treated group, however, PSD95, synaptophysin, and SNAP-25 levels were significantly higher in the hippocampus at 1 day and 4 days after ischemia compared to those in the Tat peptide or Control-SH3GL2-treated groups (Figure 5C).

## 4. Discussion

Endophilin A1 is enriched in synaptic terminals [47] and is involved in neurotransmitter release and clearance from the synaptic cleft and the morphology of synapses [23,29,48]. In the present study, we observed ischemia-dependent decreases of SH3GL2 immunoreactivity in the hippocampal CA1 region, but not in CA3 and the dentate gyrus in gerbils. To validate the role of the protein against oxidative damage in HT22 cells and ischemic damage in the gerbil hippocampus, we synthesized Tat-SH3GL2 and its control protein (Control-SH3GL2), which have no protein transduction domains. We observed that incubation with Tat-SH3GL2, not Control-SH3GL2, could be effectively delivered to HT22 cells upon 60 min of incubation at a concentrations of 0.3 μM and 0.5 μM. In addition, the Tat-SH3GL2 proteins were gradually degraded by 36 h after incubation and the proteins were visualized with polyhistidine immunohistochemical staining. To ensure the protein internalization of Tat-SH3GL2 in the HT22 cells, we observed the increases of polyhistidine levels in cell lysates and decreases in cell media. In in vivo study, we observed the Tat-SH3GL2, not Control-SH3GL2, could cross the blood–brain barrier and be delivered to the cells in the hippocampal CA1 region. This result is consistent with previous studies that showed that Tat-cargo fusion proteins could cross the membrane and had the potential to be introduced into cells or the hippocampus [17,40,41,43]. In addition, we also observed the neuroprotective effects of Tat-SH3GL2 against 1 mM H_2_O_2_-induced oxidative damage in HT22 cells by WST-1 assay. Pre-incubation of Tat-SH3GL2 prevented hippocampal neurons from oxidative stress induced by 1 mM H_2_O_2_ treatment, and we observed significant inhibitory effects by 5.0 μM Tat-SH3GL2 treatment. In the present study, we also observed that incubation with 5.0 μM Tat-SH3GL2 significantly ameliorated 1 mM H_2_O_2_-induced DNA fragmentation and ROS formation in HT22 cells. In the present study, we observed that Tat-SH3GL2 reduced ROS formation induced by 1 mM H_2_O_2_ in HT22 cells. It has been reported that *endophilin A1* Tg slices show higher susceptibility to Aβ-induced ROS formation as measured by electron paramagnetic resonance spectroscopy [33]. This discrepancy may be associated with disease models because Yu et al. [33] did not observe any significant changes in ROS formation between non-Tg and *endophilin A1* Tg slices under normal conditions. Further, in patients with Parkinson’s disease, *endophilin A1* has been reported as one of the candidate risk genes [49,50].

In the present study, we expanded our observation to an in vivo ischemic model using Mongolian gerbils. Transient forebrain ischemia showed hyperactivity 1 day after ischemia, which is an indicator of neuronal damage in the hippocampal CA1 region [17,42,51]. Treatment with Tat peptide or Control-SH3GL2 significantly increased the travel distance by more than 2.5-fold compared to that in the control group, and Tat-SH3GL2 treatment significantly ameliorated the increase in travel distance 1 day after ischemia. Consistent with this data, NeuN-immunoreactive neurons were significantly decreased in the hippocampal CA1 region of Tat peptide- or Control-SH3GL2-treated groups 4 days after ischemia because of neuronal death. We focused upon the selective neuronal death of the hippocampal CA1 region after ischemia in gerbils because endophilin A1 is observed in the hippocampal CA1 region and specific knockout of *endophilin A1* in the hippocampal CA1 region shows impairment of spatial and contextual fear memory with a similar pattern to that of whole brain knockout mice [31]. In the present study, treatment with Tat-SH3GL2 significantly increased the number of NeuN-immunoreactive neurons in the hippocampal CA1 region. This result suggests that Tat-SH3GL2 protects neurons from ischemic damage in gerbils.

To identify the possible mechanisms of Tat-SH3GL2 against ischemic damage, we observed MDA and 8-iso-PGF2α levels in the hippocampus at different times after ischemia induction because MDA and 8-iso-PGF2α are reliable markers of lipid peroxidation. Reperfusion enormously increases free radicals, which would initiate a cascade of reactions, resulting in impaired membrane function [52,53]. ROS causes the peroxidation of arachidonic acid and generates 8-iso-PGF2α in the hippocampus, and arachidonic acid has neuroprotective effects against ischemic damage induced by middle cerebral artery occlusion by reducing MDA levels in rats [54]. In addition, arachidonic acid has anti-oxidative and anti-inflammatory effects [55,56]. Treatment with Tat-SH3GL2 significantly ameliorated the increase in MDA and 8-iso-PGF2α levels in the hippocampus after transient forebrain ischemia in gerbils. In the present study, we analyzed the levels of TNF-α, IL-1β, and IL-6 in the hippocampus to validate the roles of Tat-SH3GL2 on ischemia-induced inflammatory responses because TNF-α promotes the migration of neurotrophils, IL-1β, and IL-6, which show pro-inflammatory roles. Ischemia significantly increased TNF-α, IL-1β, and IL-6 in the hippocampus and this result is supported by previous studies [46,57,58]. Treatment with Tat-SH3GL2, not Control-SH3GL2, significantly ameliorated ischemia-induced releases of TNF-α, IL-1β, and IL-6 in the hippocampus. This result suggests that Tat-SH3GL2 reduces the neuroinflammation induced by ischemia in the hippocampus. In addition, we observed PSD95, synaptophysin, and SNAP-25 in the hippocampus, which were localized in the postsynaptic and presynaptic terminals. Transient forebrain ischemia reduced PSD95, synaptophysin, and SNAP-25 levels in the hippocampus 1 day and 4 days after ischemia. This result is consistent with previous studies that revealed that these protein levels are decreased significantly in the hippocampus after ischemia [59,60,61]. In addition, exposure to oxygen-glucose deprivation significantly decreases SNAP-25 RNA in rat hippocampal neurons in primary cultures [62]. Treatment with Tat-SH3GL2 significantly mitigated the reduction of PSD95, synaptophysin, and SNAP-25 protein levels in the hippocampus 1 and 4 days after ischemia, indicating that Tat-SH3GL2 prevents the reduction of synaptic plasticity in the hippocampal CA1 region after transient forebrain ischemia.

In conclusion, Tat-SH3GL2 prevents neuronal death induced by oxidative damage in HT22 cells and ischemic damage in the gerbil hippocampus. This might be attributed to the ability of the fusion peptide to reduce oxidative damage and increased synaptic plasticity based on PSD95, synaptophysin, and SNAP-25 expression. These results suggest that Tat-SH3GL2 could be a useful neuroprotectant to reduce oxidative and ischemic damage.

## Data Availability

The datasets and supporting materials generated during and/or analyzed during the current study are available from the corresponding author on reasonable request.
